# Practical Comparison of a Newly Developed Software-Based Visual Acuity Test and Photoscreening in Japanese Preschool Children

**DOI:** 10.3390/vision10040065

**Published:** 2026-09-03

**Authors:** Yumiko Yamamoto, Timur Bakhriddinov, Hideyuki Sakaki, Narufumi Suganuma, Ichiro Murakami

**Affiliations:** 1 Yamamoto Heart and Eye Clinic, 3-1-41, Eriaecho, Nankoku 7830024, Kochi, Japan; 2 Department of Pathology, Kochi University, 185-1 Kohasu, Oko-cho, Nankoku 7838505, Kochi, Japan; ichiro-murakami@kochi-u.ac.jp; 3 Department of Environmental Medicine, Kochi University School of Medicine, Nankoku 7838505, Kochi, Japan; 4 Department of Nutritional Sciences for Well-Being, Faculty of Health Sciences, Kansai University of Welfare Sciences, 3-11-1 Asahigaoka, Kashiwara 5820026, Osaka, Japan; sakaki@tamateyama.ac.jp

**Keywords:** visual acuity testing, children, software, the Spot Vision Screener (SVS), video game

## Abstract

Background: Early detection of vision problems in young children is crucial, as they may lead to lifelong disabilities such as amblyopia and strabismus. However, conducting visual acuity (VA) evaluations in this age group is challenging. We developed novel VA testing software tailored for young children with engaging animal images and sound effects to encourage participation. Methods: A total of 414 children aged three to five years were included in the study. Each child underwent evaluation using both the newly developed VA testing software and the Spot Vision Screener (SVS, Welch Allyn, USA; software Ver. B), a portable autorefractor. Statistical analysis was performed to explore the complementary roles of VA and refractive error measurements in identifying vision problems. Results: Among the 414 children, 407 (98.3%) successfully completed the VA test using the new software. Bilateral decimal VA of 0.8 (LogMAR 0.1) or higher was observed in 383 of the 407 children (94.1%). Of the 24 children with VA < 0.8, 7 (29.2%) were already under ophthalmological follow-up, while 17 (70.8%) were newly identified as having possible visual developmental issues. SVS detected refractive errors in 21 out of 407 children (5.2%). However, 14 of the 24 children (58.3%) with VA < 0.8 showed no refractive error on SVS evaluation. Conclusions: Assessment of both VA and refractive error provides complementary information for identifying potential vision impairment in young children. Practical VA testing is particularly important, as the SVS may not detect a substantial proportion of children with possible VA deficiency. These findings support the usefulness of child-friendly VA assessment tools in preschool vision screening.

## 1. Introduction

Visual development in children typically begins around the age of three and matures by six to eight years [1,2,3,4]. Early detection and treatment of vision problems in young children are critical, as untreated conditions such as amblyopia or strabismus can lead to lifelong impairment.

In Japan, many municipalities have incorporated vision screening into annual medical check-ups conducted in kindergartens. However, teachers and nurses involved in these screenings often lack specialized training in visual acuity (VA) evaluation [2]. Moreover, equipment designed specifically for pediatric VA assessment remains limited, posing challenges in obtaining accurate results for this age group. To address these limitations, we developed novel vision testing software tailored for children aged 3 to 5 years. In Japan, VA is routinely assessed using decimal VA levels with the Landolt-C optotype, which is the standardized and legally mandated optotype for clinical and educational screening [5]. Given this context, the software calculates Landolt-C ring sizes based on a proprietary algorithm in accordance with ISO standards [6].

In this study, we conducted VA assessments using the newly developed software, together with refractive error evaluations performed by the Spot Vision Screener (SVS, Welch Allyn, USA; software Ver.B), to examine their complementary roles in pediatric vision screening.

## 2. Materials and Methods

### 2.1. Newly Developed VA Testing Software

**Figure 1:**
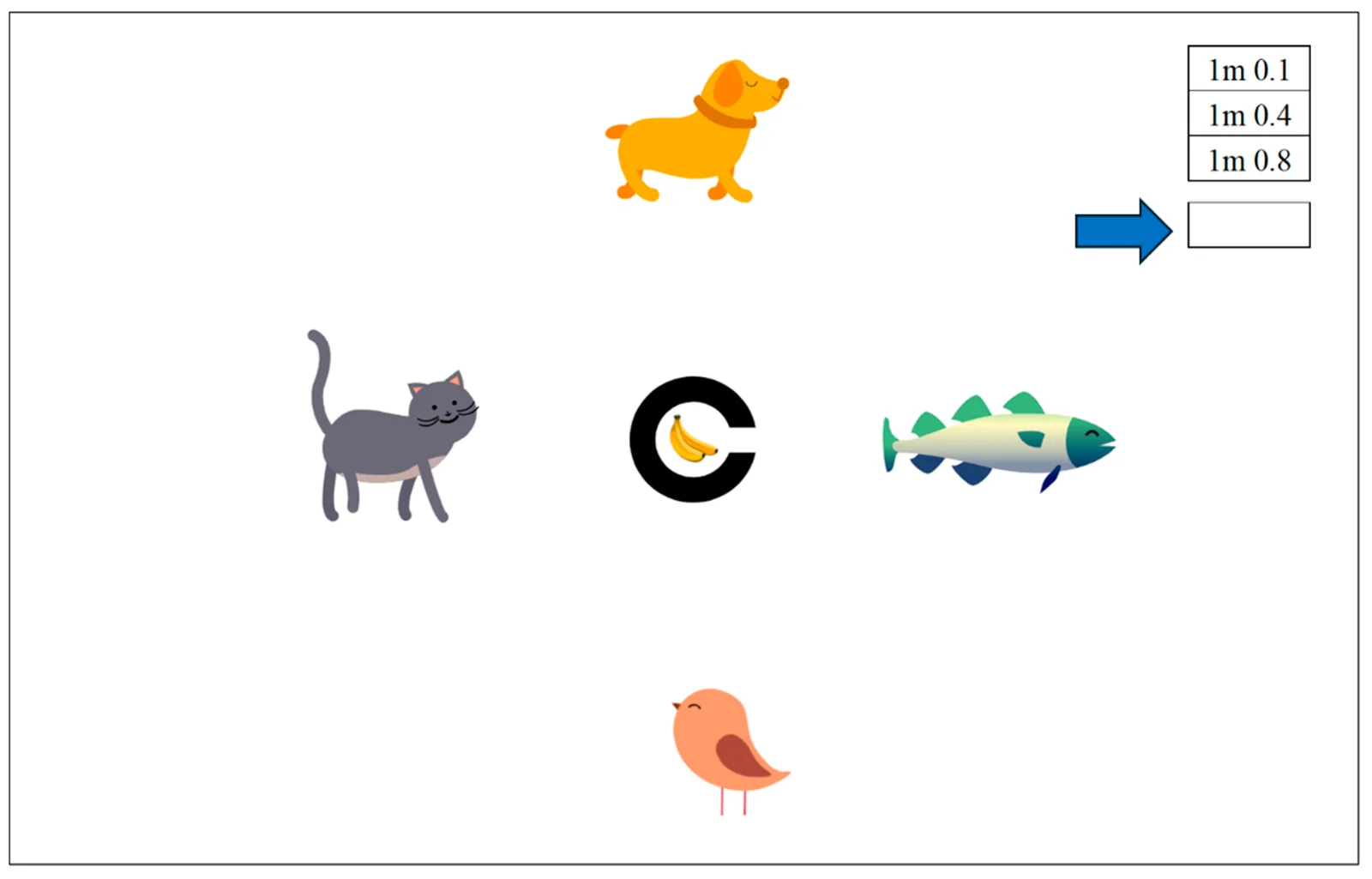
Screenshot of the VA Testing software interface. Clicking on one of the visual acuity level buttons (0.1, 0.4, or 0.8; upper right) automatically adjusts the Landolt-C ring size according to international standards. In the 0.1 assessment only, a banana image is displayed inside the Landolt-C ring to help children understand the task. For the 0.4 and 0.8 assessments, no image appears inside the ring, and the banana animation is shown only after the child’s response, pumping out through the slit toward one of four animal characters (dog, cat, bird, or fish). The numeric value required for calibration is entered in the designated field highlighted by the blue arrow. Animations and sound effects help maintain children‘s attention, and the test is designed to be language- and literacy-independent. The program runs on any modern PC and can be administered without prior medical training.

A new VA testing software was collaboratively developed by an experienced ophthalmologist (YY) and a professional web designer (TB). The program integrates engaging animations and sound effects resembling a video game interface [7]. The testing screen features four animated animals—dog, cat, bird, and fish—and a Landolt-C ring displaying a banana image that shifts toward one of the animals through the ring’s slit. The Landolt-C ring size is automatically configured to international standards based on predefined decimal VA levels (e.g., 0.1, 0.4, and 0.8, corresponding to LogMAR 1.0, 0.4, and 0.1) [6]. A schematic illustration of the software interface is provided in Figure 1, intended to show the characters, icons, and interface elements used in the software. This figure is not an actual test screen but an explanatory diagram with enlarged elements and an arrow indicating the calibration-input field. The examination may be conducted at adjustable distances such as 1, 3, and 5 m. After the testing distance is selected, a corresponding numeric value is entered in the calibration field (highlighted by the blue arrow in Figure 1) to adjust the ring’s dimensions. Because the Landolt-C ring scales uniformly, the examiner simply selects a numeric value that makes the 0.1-level ring size, the largest optotype, close to its theoretical dimensions for the chosen testing distance (e.g., 14.5 mm diameter at one meter, Table 1), using a standard ruler on the screen. This calibration does not require any special skill. Although simple, both parameters (the diameter and slit width) are adjusted with sub-millimeter precision because they change simultaneously, allowing accurate calibration. Once this single calibration is completed for a given monitor and testing distance, the same value can be reused consistently, and no further adjustment is needed. It remains stable across repeated software launches and computer restarts. The interface allows simple click-based interactions, while the software automatically randomizes the ring direction at the examiner-selected VA level. Literacy is not required to complete the assessment.

### 2.2. Participants

This study included 414 children aged 3 to 5 years attending five kindergartens in Nankoku City, Kochi, Japan: four public institutions and one private institution. Participants comprised 127 three-year-olds, 138 four-year-olds, and 149 five-year-olds. The study was conducted with full cooperation from the respective principals and the city’s Children Support Center, following prior explanation of the methodology and anticipated benefits.

### 2.3. Prior to the Tests

Before testing, children played a warm-up game with their teachers using cards that matched the visual patterns of the actual screen—featuring animals and a Landolt-C ring with a banana at its center. Through this activity, they learned the rules of the test: to identify which animal would receive the banana as it could slide through the slit of the ring. As they grew familiar with the task, they also learned to play while covering one eye, naturally understanding that the test was performed monocularly.

### 2.4. Performance of the Test

**Figure 2:**
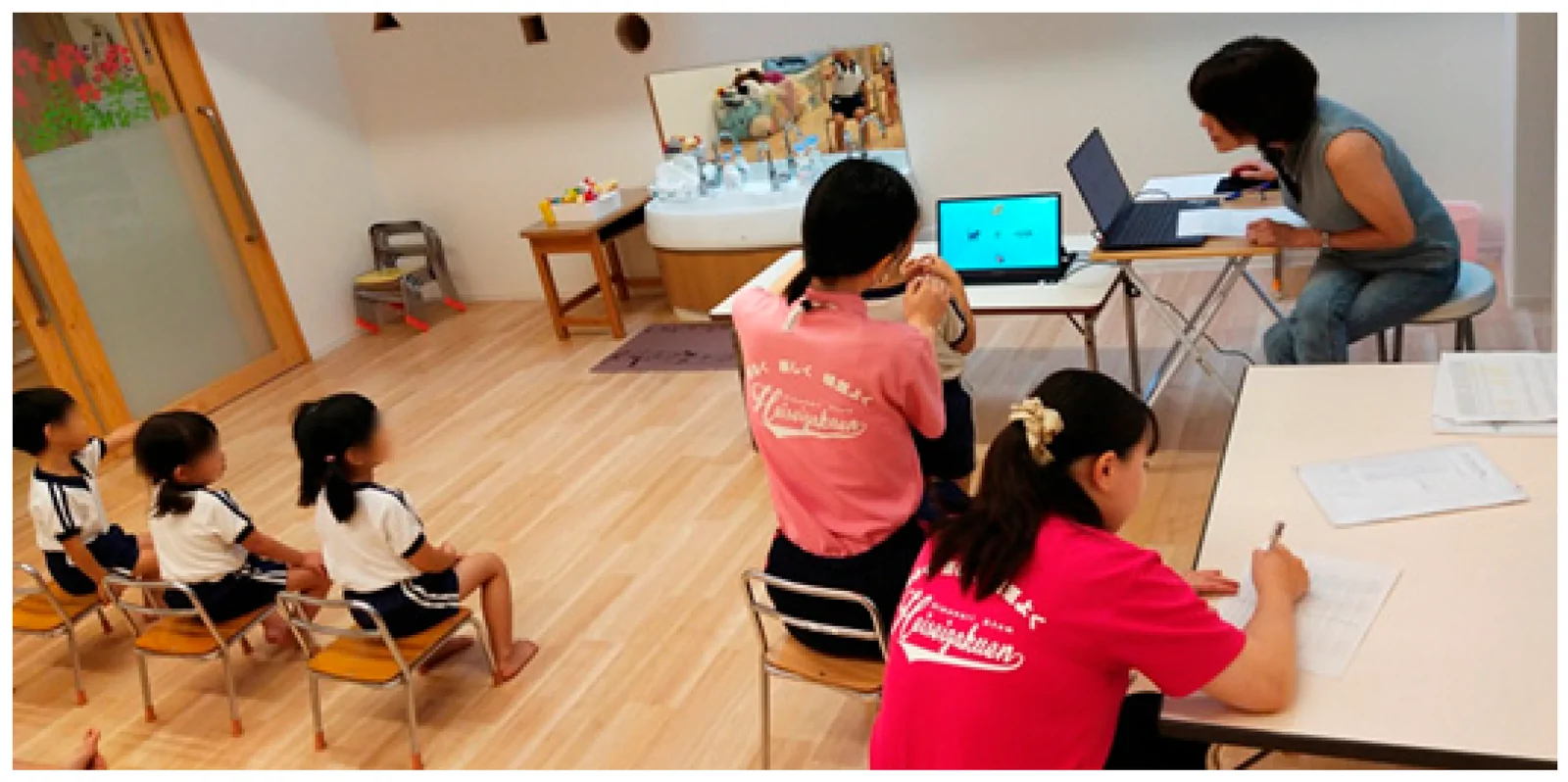
Vision Screening at a Kindergarten. Monocular vision testing was conducted at a one-meter distance from a monitor. The non-tested eye was manually occluded by a teacher seated behind the child. The examiner operated the software using simple mouse clicks and observed whether the occlusion was effective and whether the child was attentively engaging with the test screen. Faces of pediatric participants are blurred for privacy. The examiner shown is the first and corresponding author (Yumiko Yamamoto), appearing with consent. A representative video of the procedure is provided as Supplementary Video S1.

Each child stood at a one-meter distance from the screen to receive the VA test. The VA of each eye was evaluated while the opposite eye was gently covered by the teacher’s hand; the teacher sat behind the child to provide physical and emotional support. Children awaiting their turn sat behind, observing the examination process. VA levels of 0.1, 0.4, and 0.8 were assessed based on the standard clinical procedures described in Section 2.5, with all measurements performed on a single device by an experienced ophthalmologist (YY), who operated the mouse and conducted the evaluation. The examiner observed the children carefully throughout the process, ensuring that each eye was properly covered and that the child’s gaze remained focused on the screen. The procedure is illustrated in Figure 2 and Supplementary Video S1, which shows real-time interaction and testing as described.

### 2.5. Evaluation of the VA Level

VA level was determined when a child successfully identified the correct direction of the Landolt-C ring three times, consistent with standard clinical practice. The cutoff of 0.8 was selected based on the Japanese Ophthalmological Society’s screening manual [8]. In cases where the measured VA was below 0.8, whether due to reduced VA or potential comprehension or concentration issues, the test was repeated on another day, and the highest score was selected as the final evaluation result. Corrected VA was not assessed, except for children who were already wearing glasses, as the examination was performed as a screening within the limited time frame allocated by each kindergarten.

### 2.6. Evaluation of the Refractive Error

The refractive error examination was conducted using the SVS by a teacher at each participating kindergarten, who had received a brief orientation on how to operate the device. Refractive errors were identified based on diagnostic criteria pre-installed in the SVS. If a refractive error was detected, an additional measurement was performed on the same day or an alternative day. A reproducible result was adopted as the final value. Although the SVS also offers simultaneous strabismus screening, this study focused specifically on the evaluation of refractive error and VA. However, when strabismus was incidentally detected, the result was shared with the child’s parents.

### 2.7. Statistical Analysis

Vision test results—including evaluations of VA and refractive error measured by the newly developed software and the SVS, respectively—were analyzed alongside clinical background data such as age and prior episodes related to visual problems. A chi-square test was used to compare the incidence of VA below 0.8 across three age groups and five kindergartens. The number of testing sessions was not evaluated, because repeated testing occurred for random, non-scientific reasons, such as momentary emotional or behavioral fluctuations, unrelated to children’s visual function.

### 2.8. IRB Approval and Informed Consent

This study was approved by the Ethical Committee of Kochi Medical School, Kochi University (Approval No. 2023-3). Written informed consent to participate was obtained from each child’s parent, who also completed a questionnaire about the child’s vision and eye health. All personal information was strictly protected and used solely for research purposes. Results and examiner comments were shared with parents of children who might require further assessment or follow-up at an eye clinic. Additionally, parents who submitted inquiries or requested individual results received written feedback, regardless of whether any abnormal findings were detected.

## 3. Results

VA was successfully measured using the newly developed software in 407 out of 414 participating children (98.3%) (Table 2). Seven children (four aged three years and three aged four years) were unable to complete the test due to miscomprehension or difficulty maintaining concentration. Apart from these seven children, all participants were able to recognize the 0.1-level optotype. Among the 407 children who completed the test, 383 (94.1%) had a VA of 0.8 or higher in both eyes. Conversely, 24 children (5.9%) were identified as having VA below 0.8 in either or both eyes. Of these 24 children, only 7 (29.2%) had previously visited an ophthalmology clinic for prescription of glasses or other clinical eye evaluations. The remaining 17 children (70.8%) had not been identified as potentially having poor visual development.

**Figure 3:**
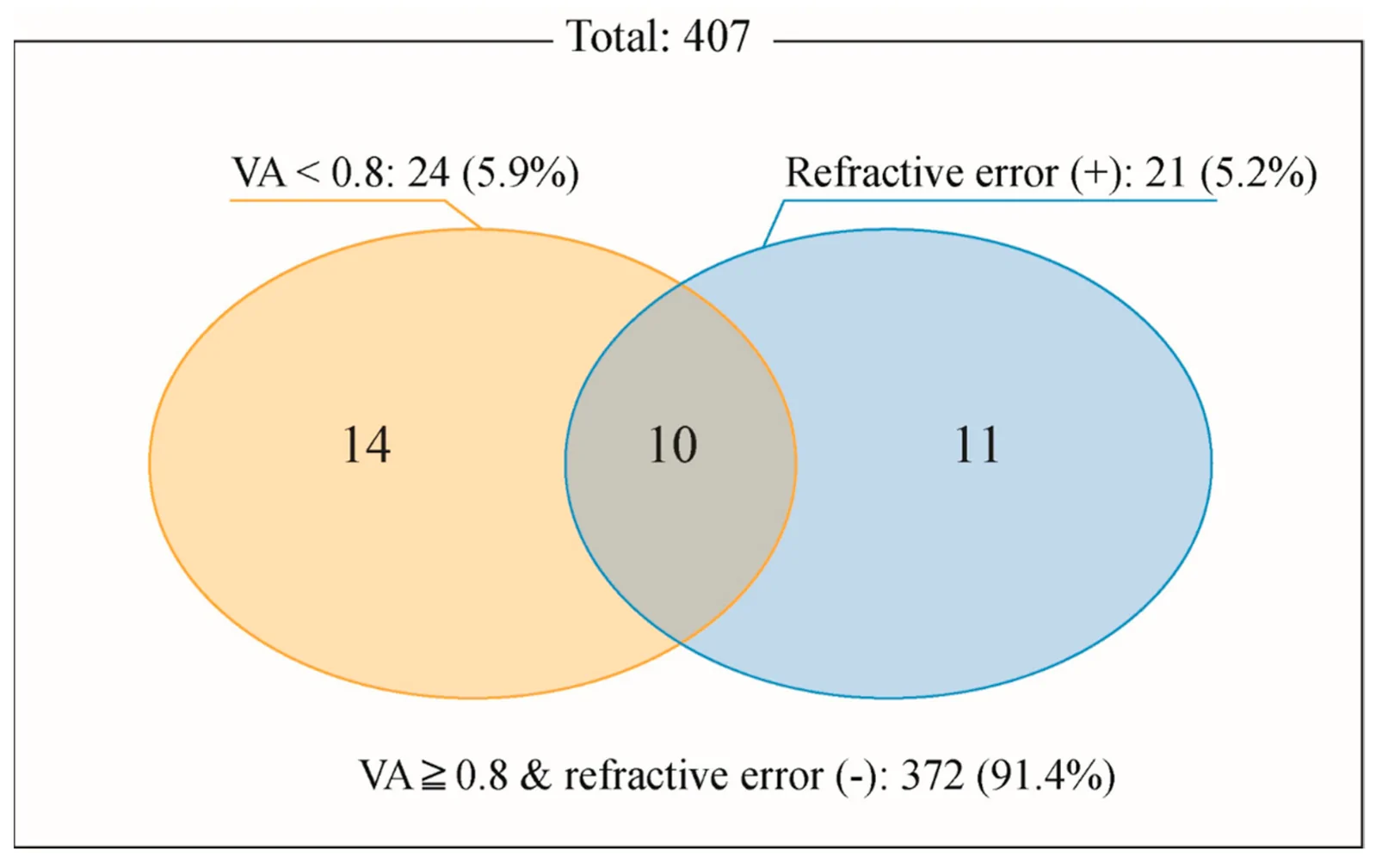
Overlap of Refractive Conditions among Participants. Venn diagram depicting the overlap between two screening outcomes: VA < 0.8 and refractive errors detected by the Spot Vision Screener. Each circle represents participants meeting one criterion, and the overlapping region identifies individuals who met both conditions.

Refractive error assessment using the SVS detected refractive errors in 21 of 407 children (5.2%), with specific error messages listed in Table 3. Among the 24 children with VA below 0.8, 14 (58.3%) were not flagged by the SVS as having refractive errors (Figure 3). No statistically significant differences in the incidence of VA below 0.8 or refractive error were observed among the three age groups or five kindergartens (chi-square test).

Responses to the three questions in the questionnaire (Supplementary Materials) varied widely among parents. Comments included concerns about overuse of digital devices, paternal color vision deficiency, and issues including trichiasis, allergies, or mild tic symptoms. Although each comment was addressed individually by the ophthalmologist (the first author), they were not statistically related to the vision screening findings in this study. In addition, information regarding the exact number or reasons for the approximately five parents who did not agree to their children’s participation was not shared with the research team. These parental decisions reflected individual personal or private circumstances.

## 4. Discussion

In 1991, the Japanese government introduced a vision test at the municipal 3.5-year-old health check-up [8,9,10]. According to the national guidance, the primary VA test is conducted by parents at home, an approach that lacks clinical reliability [2]. At the check-up center, secondary VA tests are occasionally requested, typically only for children whose parents express concern. This may result in underdiagnosis by missing the opportunity to evaluate children who may need further assessment. Even when secondary testing is offered, the absence of child-specific tools and insufficient training among nurses at check-up centers present significant barriers to reliable VA screening in young children [2].

Several digital tools, such as Peekaboo Vision and Kay iSight Pro, currently represent the state of the art in early childhood vision screening in several countries [11,12]. However, they are not popular in Japan, partly because VA evaluation in Japan is based on the exclusive use of Landolt-C optotypes for official assessment. In addition, Japanese-language versions of these tools are not readily available, and their adoption in routine preschool or school screening programs has therefore remained limited. Nevertheless, it is generally considered that the limited domestic use of these tools does not diminish their relevance as part of the international state of the art. Our software contributes to this landscape by providing a standardized Landolt-C–based method, offering compatibility with Japanese VA evaluation standards.

In recent years, Japanese municipalities have introduced the SVS at the 3.5-year-old health check-up. The SVS is a widely used international tool for detecting ametropia, anisometropia, and strabismus in children [10,13,14,15,16]. Though the SVS was considered a promising advance in vision screening for children, concerns have been raised regarding its relatively low sensitivity, particularly for hyperopia [10,13,16,17,18,19,20,21]. Previous studies recommend cycloplegic refraction for greater diagnostic accuracy [17,20]. Voide et al. reported that only 39.3% of children with visual abnormalities were identified by SVS, compared to 60.7% through comprehensive clinical examination [18]. Matsuo et al., in a study involving 265 children, concluded that SVS autorefraction was not cost-effective as an additional tool within the current Japanese check-up system [19]. As a result, despite the introduction of SVS, the current municipal protocols still lack pathways to identify and evaluate children who may require a further VA evaluation [19]. Consequently, some children are diagnosed with poor vision during only later clinical encounters or at the preschool health screening conducted prior to school entry. To address the lack of vision screening between the 3.5-year health check-up and the school-entry health screening, some local governments have implemented mandatory VA testing in kindergartens. Nonetheless, barriers remain: teachers and nurses lack specialized training, and no universally practical tools exist for VA screening in young children. Recognizing this practical limitation in early childhood environments—including kindergartens and municipal screening venues, we developed novel VA testing software specifically tailored to children aged 3 to 5. In our study involving 414 participants, we found a high completion rate and strong practical utility, demonstrating feasibility for broader use by non-specialist personnel with appropriate guidance or training.

The simplified online version of the software is freely accessible at https://vision-test.vercel.app/, accessed on 18 May 2026, as it was developed to support early childhood vision screening and promote equitable access to eye health tools. Full-featured versions (for both Windows and macOS) are available upon request from the authors. The software was designed for ease of installation and operation, requiring no special skills for use. Its flexible calibration system allows testing at various distances—one, three, or five meters—adapting to facility space and examination preferences. For example, testing from a greater distance is ideal during infectious outbreaks, whereas closer observation may be preferable in kindergartens or 3.5-year-old check-up centers. When used under appropriate lighting conditions (approximately 300–500 lux) and resolution, and in adherence to standard VA testing protocols, the software reliably presents calibrated optotype charts. Because its Landolt-C ring sizing algorithm conforms to conventional charts, results are clinically reproducible [5]. The software’s independence from language-based comprehension further supports its adaptability in diverse global settings. The software also supports near-vision testing at 0.3-m or 0.5-m distances (Supplementary Table S1), which may help identify signs of hyperopia—a condition that affects approximately 8–10% of young children and may impair learning and development progress if left undetected [4,22,23,24].

In this study, the participants were young children between three and five years of age, and VA evaluation was more easily and effectively performed at a close distance (one meter), allowing the examiner to observe the children’s responses with greater precision. Children were accompanied by their teacher sitting behind them, who gently covered the lateral eye with their hand. This approach helped keep the children calm and comfortable, while providing more stable covering than materials such as metal, plastic, or paper. In Japanese kindergartens, close physical contact between teachers and children is common and culturally accepted. However, for use in international settings, non-contact methods for occluding one eye would need to be considered. Additionally, almost all children in this study were undergoing VA testing for the first time, and some showed unstable attention or understanding during the initial attempt. Therefore, we adopted the better result from repeated testing to minimize misclassification due to transient inattention during the first attempt. Repeated attempts occurred in approximately 30% of 3-year-olds and 17% of 4–5-year-olds. During the repeated attempt, most children found that the test was fun and easy, like a video game, and were more comfortable, focused, and cooperative. Such practice effects are unavoidable in pediatric ophthalmic testing, representing a routine and expected aspect of clinical practice rather than a limitation of the present study.

Among 407 children, 24 (5.9%) were evaluated with VA less than 0.8. 17/24 (70.8%) children had no prior vision-related episodes noted by parents or municipal screenings. Additionally, 14/24 (58.3%) were not flagged for refractive errors by the SVS. These results demonstrate that SVS alone may be insufficient for identifying children with possible delayed visual development. As the previous literature supports [17,18,19,20,21], VA and refractive error represent distinct aspects of vision; thus, incorporating practical VA testing with the SVS enhances overall screening accuracy by integrating subjective and objective viewpoints. Vision screening in children should not rely solely on the refractometer but should also include a practical VA evaluation. Practical VA testing complements refractometry, enhances screening accuracy, and helps to identify children who may otherwise remain undetected.

Despite these valuable insights and advances, several practical challenges persist. While all VA tests in this study were conducted by a single examiner to eliminate variability, future developments will involve teachers and nurses to broaden implementation. To strengthen clinical relevance, diagnostic accuracy will be further explored by comparing outcomes of the VA result using the software with clinical evaluations. Preparations for the required IRB approval for this subsequent study are currently underway, which will address current limitations and inform future validation efforts.

## 5. Conclusions

The novel software achieved a high completion rate of 98.3% in VA evaluation among Japanese children aged three to five years, highlighting its practical feasibility and screening utility. Among children with VA less than 0.8, 58.3% (14/24) showed no refractive error according to SVS. These findings underscore the importance of incorporating practical VA testing into pediatric screening protocols, as refractometry alone may fail to detect cases needing further examination.

## Figures and Tables

**Table 1:** Theoretical Dimensions of Ring Optotypes for VA testing from 1 m. This table presents the decimal and logMAR visual acuity (VA) values, along with the calculated diameters and slit widths of Landolt-C ring optotypes used for VA testing at 1 m in the study.

Desimal VA	LogMAR VA	Diameter of the Ring (mm)	Slit of the Ring (mm)
1.0	0.000	1.5	0.3
0.8	0.097	1.8	0.4
0.4	0.398	3.6	0.7
0.1	1.000	14.5	2.9

**Table 2:** Summary of Vision Screening Outcomes by Age Group. The table presents visual acuity and refractive error data for children aged 3 to 5 years. Among the 407 examined children, 94.1% achieved bilateral visual acuity of 0.8 or higher, while 5.2% showed signs of refractive error.

	N: Total	N: NA	N: Examined	N: Bilateral VA ≧ 0.8		Refractive Error (+)	
3 years old	127	4	123	114	92.7%	5	4.1%
4 years old	138	3	135	124	91.9%	7	5.2%
5 years old	149	0	149	145	97.3%	9	6.0%
Total	414	7	407	383	94.1%	21	5.2%

**Table 3:** Distribution of refractive status among particcipants. Number and proportion of participants classified by refractive condition, including myopia, astigmatism, myopic astigmatism, and emmetropia.

Refractive Status	n	%
Myopia	6	14.3%
Astigmatism	27	64.3%
Myopic Astigmatism	1	2.4%
Emmetropia	8	19.0%
Total	42	100.0%

## Data Availability

The original contributions presented in this study are included in the article/Supplementary Materials. Further inquiries can be directed to the corresponding author(s).

## Supplementary Materials

The following supporting information can be downloaded at: https://www.mdpi.com/article/10.3390/vision10040065/s1, Table S1: Theoretical Dimensions of Ring Optotypes for VA testing from 5 m to 0.3 m; Video S1: Experimental Operation Video.

## References

[1] Myklebust A.K., Riddell P.M. (2022). Development of visual acuity in children: Assessing the contributions of cognition and age in LEA chart acuity readings. Optom. Vis. Sci..

[2] Osborne D., Steele A., Evans M., Ellis H., Pancholi R., Harding T., Dee J., Leary R., Bradshaw J., O’Flynn E. (2023). Children’s visual acuity tests without professional supervision: A prospective repeated-measures study. Eye.

[3] Mills M.D. (1999). The eye in childhood. Am. Fam. Physician.

[4] Nunes A.F., Sena F., Calado R., Tuna A.R.R., Gonçalves A.P.R., Monteiro P.L. (2021). Reduced visual acuity in children aged 5–6 years using the LEA chart. Graefes Arch. Clin. Exp. Ophthalmol..

[5] Visual Acuity. https://www.hc.u-tokyo.ac.jp/en/checkupresult/explanation/va/.

[6] (1984). Visual Acuity Measurement Standard.

[7] Bodduluri L., Boon M.Y., Ryan M., Dain S.J. (2017). Impact of Gamification of Vision Tests on the User Experience. Games Health J..

[8] Japan Ophthalmologists Association: Tokyo, Japan. https://www.gankaikai.or.jp/school-health/2021_sansaijimanual.pdf.

[9] Hayashi S., Suzuki I., Inamura A., Iino Y., Nishitsuka K., Nishina S., Yamashita H. (2021). Effectiveness of the Spot Vision Screener in screening 3-year-old children with potential amblyopia in Japan. Jpn. J. Ophthalmol..

[10] Matsuo T., Matsuo C., Kayano M., Mitsufuji A., Satou C., Matsuoka H. (2022). Photorefraction with Spot Vision Screener versus visual acuity testing as community-based preschool vision screening at the age of 3.5 years in Japan. Int. J. Environ. Res. Public Health.

[11] Peekaboo Vision—Digital Infant Vision Testing. https://www.peekaboo.scot.nhs.uk/.

[12] The Kay iSight Pro App: A Versatile Tool for Cost-Effective Eye Testing Across All Ages. https://kaypictures.co.uk/2024/10/24/the-kay-isight-pro-app/.

[13] Peterseim M.M.W., Trivedi R.H., Monahan S.R., Smith S.M., Bowsher J.D., Alex A., Wilson M.E., Wolf B.J. (2023). Effectiveness of the Spot Vision Screener using updated 2021 AAPOS guidelines. J. AAPOS.

[14] Gaiser H., Moore B., Srinivasan G., Solaka N., He R. (2020). Detection of amblyogenic refractive error using the Spot Vision Screener in children. Optom. Vis. Sci..

[15] Peterseim M.M.W., Trivedi R.H., Feldman S., Husain M., Walker M., Wilson M.E., Wolf B.J. (2020). Evaluation of the Spot Vision Screener in school-aged children. J. Pediatr. Ophthalmol. Strabismus.

[16] American Association for Pediatric Ophthalmology and Strabismus (2021). Vision Screening Recommendations.

[17] Barugel R., Touhami S., Samama S., Landre C., Busquet G., Vera L., Quoc E.B. (2019). Evaluation of the Spot Vision Screener for children with limited access to ocular health care. J. AAPOS.

[18] Voide N., Hoeckele N., Kaeser P.F. (2018). Comparative study of the usefulness of the binocular Spot Vision Screener autorefractor in detecting childhood visual disorders. Klin. Monatsblätter Augenheilkd..

[19] Matsuo T., Matsuo C., Kio K., Ichiba N., Matsuoka H. (2009). Is refraction with a handheld autorefractometer useful in addition to visual acuity testing and questionnaires in preschool vision screening at 3.5 years in Japan?. Acta Med. Okayama.

[20] Nelson L.B. (2021). The role of the Spot Vision Screener. J. Pediatr. Ophthalmol. Strabismus.

[21] Kapoor V., Shah S.P., Beckman T., Gole G. (2022). Community-based vision screening in preschool children: Performance of the Spot Vision Screener and optotype testing. Ophthalmic Epidemiol..

[22] Khoshhal F., Hashemi H., Hooshmand E., Saatchi M., Yekta A., Aghamirsalim M., Ostadimoghaddam H. (2020). The prevalence of refractive errors in the Middle East: A systematic review and meta-analysis. Int. Ophthalmol..

[23] Castagno V.D., Fassa A.G., Carret M.L.V., Vilela M.A.P., Meucci R.D. (2014). Hyperopia: A meta-analysis of prevalence and a review of associated factors among school-aged children. BMC Ophthalmol..

[24] Hopkins S., Read S.A., Cox R.A., Oduro B.A., Strang N., Wood J.M. (2024). Hyperopia in schoolchildren: Investigating the impact on vision and determining appropriate methods for screening. Ophthalmic Physiol. Opt..

