# Supplementary material for: Practical Comparison of a Newly Developed Software-Based Visual Acuity Test and Photoscreening in Japanese Preschool Children
Source: Vision (Basel). 2026 Sep 3;10(4):65. doi: 10.3390/vision10040065 (PMC13600185; doi:10.3390/vision10040065)
Supplement: Supplementary file 1 [file vision-10-00065-s001.zip › Questionnaire.zip!/Questionnaire.pdf]

To Guardians

Dr. Yumiko Yamamoto, the Lecturer (Ophthalmologist) at Kochi University Hospital, has initiated a research study on visual acuity screening for preschool children. The study aims to detect potential visual development issues before school entry. Currently, there is no standardized method for testing visual acuity in children aged 3–5 who may not fully understand the purpose of such tests. This research uses animation-based software designed to make the testing experience enjoyable and game-like for young children. The test will be conducted by Dr. Yamamoto during regular childcare hours at the kindergarten and will take approximately five minutes per child. Results will be shared with you later. Participation is voluntary, and each guardian of children may decline or withdraw consent at any time by notifying the homeroom teacher. This study aims to support early detection of visual issues in preschool children. Even if your child participates, no personal information will be disclosed outside the research team.

If you have any questions, please write them below. Dr. Yamamoto will respond directly.

++

Consent Section

1. I wish / do not wish my child to participate in the visual acuity test. (If you choose "No," you do not need to answer the questions below.)
2. Have you ever had concerns about your child's eyes?
   Yes / No (If yes, please specify:)
3. Has your child ever been diagnosed with any eye condition?
   No / Yes (If yes, please specify:)
4. Additional Questions
   No / Yes (If yes, please specify:)

Child's Name (with phonetic spelling): ____________ year ____ class ____

Your Name: ____________________ Date: ____________
